# The new IL-1 family member IL-1F8 stimulates production of inflammatory mediators by synovial fibroblasts and articular chondrocytes

**DOI:** 10.1186/ar1946

**Published:** 2006-04-28

**Authors:** David Magne, Gaby Palmer, Jenny L Barton, Francoise Mézin, Dominique Talabot-Ayer, Sylvette Bas, Trevor Duffy, Marcus Noger, Pierre-Andre Guerne, Martin JH Nicklin, Cem Gabay

**Affiliations:** 1 Division of Rheumatology, Department of Internal Medicine, University Hospital and Department of Pathology and Immunology, University of Geneva School of Medicine, Geneva, Switzerland; 2 Division of Genomic Medicine, University of Sheffield, Henry Wellcome Laboratories for Medical Research, Medical School, Sheffield, UK; 3 Department of Orthopedic Surgery, University Hospital of Geneva, Geneva, Switzerland

## Abstract

Six novel members of the IL-1 family of cytokines were recently identified, primarily through the use of DNA database searches for IL-1 homologues, and were named IL-1F5 to IL-1F10. In the present study, we investigated the effect of IL-1F8 on primary human joint cells, and examined the expression of the new IL-1 family members in human and mouse joints. Human synovial fibroblasts (hSFs) and human articular chondrocytes (hACs) expressed the IL-1F8 receptor (IL-1Rrp2) and produced pro-inflammatory mediators in response to recombinant IL-1F8. IL-1F8 mRNA expression was increased in hSFs upon stimulation with proinflammatory cytokines, whereas in hACs IL-1F8 mRNA expression was constitutive. However, IL-1F8 protein was undetectable in hSF and hAC culture supernatants. Furthermore, although IL-1β protein levels were increased in inflamed human and mouse joint tissue, IL-1F8 protein levels were not. IL-1F8 levels in synovial fluids were similar to or lower than those in matched serum samples, suggesting that the joint itself is not a major source of IL-1F8. Serum levels of IL-1F8 were similar in healthy donors, and patients with rheumatoid arthritis, osteoarthritis and septic shock, and did not correlate with inflammatory status. Interestingly however, we observed high IL-1F8 levels in several serum samples in all groups. In conclusion, IL-1F8 exerts proinflammatory effects in primary human joint cells. Joint and serum IL-1F8 protein levels did not correlate with inflammation, but they were high in some human serum samples tested, including samples from patients with rheumatoid arthritis. It remains to be determined whether circulating IL-1F8 can contribute to joint inflammation in rheumatoid arthritis.

## Introduction

Until recently, the IL-1 family of cytokines included four members, with three having pro-inflammatory effects (IL-1α, IL-1β and IL-18) and the fourth member being an IL-1 receptor antagonist (IL-1Ra). IL-1 family members exert their effects through binding to receptors that belong to the IL-1 receptor (IL-1R) family. IL-1α and IL-1β bind to the type I IL-1 receptor (IL-1RI), resulting in recruitment of the IL-1 receptor accessory protein (IL-1RAcP), which is necessary for signal transduction. IL-1Ra negatively regulates IL-1 activity by competing with IL-1 for binding to IL-1RI. Binding of IL-1Ra to IL-1RI does not allow the recruitment of the accessory protein, and therefore it does not generate a signal (for review see [1]). IL-18 activity is mediated through its binding to other members of the same receptor family, namely IL-18 receptor (IL-18R) and the IL-18R accessory protein [2].

Six new members of the IL-1 family were recently identified, primarily through the use of DNA database searches for homologues of IL-1 [3-10]. These proteins were named IL-1F5 to IL-1F10 [11]. In humans all of the new genes map to less than 300 kb of chromosome 2, where they are flanked by IL-1α, IL-1β and IL-1Ra. Sequence alignments and some physical data predict that the secondary structure of all of the new homologues is characterized by a 12-stranded β-trefoil structure shared with IL-1α, IL-1β and IL-1Ra [12]. IL-1F5 was recently characterized at high resolution [13].

Expression patterns and the biological functions of the six new IL-1 family members have not yet been well characterized. It has been reported that IL-1F7 forms a complex with IL-18 binding protein, which might bind to and sequester IL-18R accessory protein, thus inhibiting the effects of IL-18 [14]. In addition, adenoviral overexpression of IL-1F7 in mouse was shown to have anti-tumour effects by an undefined mechanism, even though rodents appear to lack the IL-1F7 gene [15]. IL-1F10 has been described as a low affinity, nonagonistic ligand for IL-1RI [7]. Debets and coworkers [5] have shown that IL-1F9 activates nuclear factor-κB in Jurkat cells that overexpress IL-1 receptor related protein 2 (IL-1Rrp2) and that this activation is blocked by IL-1F5, suggesting that IL-1F5 might be an IL-1F9 antagonist. Recently, Towne and coworkers [16] reported that, in addition to IL-1F9, IL-1F6 and IL-1F8 also activated nuclear factor-κB and showed that signalling required IL-1RAcP. Inhibition of IL-1F6-, IL-1F8-, or IL-1F9-mediated activation of nuclear factor-κB by IL-1F5 was described as incomplete and inconsistent. In that study, using an epithelial cell line that expresses both IL-1Rrp2 and IL-1RAcP, the three homologues activated an IL-8 promoter reporter gene construct and secretion of IL-6, even though the required IL-1F concentrations were much higher than those necessary for IL-1β activity.

Rheumatoid arthritis (RA) is characterized by chronic inflammation of the synovial tissue in multiple joints that leads to joint destruction. Major hypotheses have involved dysfunction of antigen-presenting cells; B cells and autoantibody production; T cell reactivity; and, recently, cytokines (for review, see [17]). Indeed, it is widely recognized that tumour necrosis factor (TNF)-α and IL-1 play key roles in mediating the pathophysiological processes that underlie the inflammation and tissue destruction that occur in RA. The role of the four 'classical' IL-1 family members (for instance, IL-1α, IL-1β, IL-1Ra and IL-18) in the pathogenesis and development of RA was illustrated in mouse models of arthritis, particularly by the spontaneous arthritis that develops in IL-1α transgenic mice [18] as well as in IL-1Ra deficient mice [19]. It was also highlighted by the significant protection against collagen-induced arthritis (CIA) that characterizes overexpression of IL-1Ra [20,21] and genetic deficiency in IL-1α, IL-1β [22], or IL-18 [23].

In the present study we investigated the effects of the new IL-1 family member IL-1F8 on primary human synovial fibroblasts (hSFs) and human articular chondrocytes (hACs), and examined the expression of the new IL-1 homologues in human and mouse joints.

## Materials and methods

### Materials

Cell culture reagents were obtained from Invitrogen Life Technologies (Basel, Switzerland). Recombinant human IL-1β, recombinant human IL-1F8 and goat polyclonal anti-human IL-1Rrp2, as well as anti-human and anti-mouse IL-1F8 antibodies, were purchased from R&D Systems (Abington, UK). Trizol reagent and dNTP were obtained from Invitrogen. Taq DNA polymerase was obtained from Qiagen AG (Basel, Switzerland). DNase I, AMV-RT (avian myeloblastosis virus-reverse transcriptase), random primers, recombinant ribonuclease inhibitor and DNA 100 bp ladder were purchased from Promega (Wallisellen, Switzerland). DNA Master SYBR green I or Fast Start DNA Master SYBR green I kits were obtained from Roche Molecular Biochemicals (Rotkreuz, Swizerland).

### Cell culture

Synovium and articular cartilage were obtained from patients undergoing joint replacement (knee or hip prosthetic surgery) for osteoarthritis (OA) or broken femoral neck (normal adult articular cartilage). hSFs and hACs were isolated by collagenase digestion, as reported previously [24], and cultured in Dulbecco's modified Eagle medium supplemented with l-glutamine, streptomycin, penicillin and 10% heat-inactivated foetal calf serum (FCS) at 37°C in a humidified atmosphere containing 5% CO_2_. Primary hACs were used directly after isolation from cartilage and hSFs were used between passages 2 and 8. To reduce the nonspecific effects of agonists present in FCS, cells were incubated overnight in low-serum (0.5% FCS) medium before the various treatments.

### RNA isolation

For RNA isolation, hSFs and hACs were seeded in 25 or 75 cm^2 ^flasks. After the indicated incubation times, media were removed and cells were lyzed in Trizol. Total RNA was prepared according to the manufacturer's instructions. Briefly, homogenization of tissues in Trizol was followed by centrifugation at 10,000 rpm (4°C) for 15 minutes in the presence of chloroform. The upper aqueous phase was collected and total RNA was precipitated by addition of isopropanol and centrifugation at 7,500 rpm (4°C) for 5 minutes. RNA pellets were washed with 75% ethanol, dried, reconstituted with sterile water and quantified by spectrometry.

### Reverse transcription and polymerase chain reaction

For analysis of mRNA levels by RT-PCR and real-time PCR, 1–3 μg total RNA were used. After DNase I digestion, RNA samples were reverse transcribed using AMV-RT (avian myeloblastosis virus-reverse transcriptase) and random primers in a total volume of 30–50 μl. Template cDNAs (2.5 μl) were amplified in a typical 25 μl PCR reaction containing 20 mmol/l Tris-HCl (pH 8.4), 50 mmol/l KCl, 1 μmol/l of the respective primers (Table 1), 2 mmol/l MgCl_2_, 200 μmol/l dNTP and 2.5 units Taq DNA polymerase. The absence of DNA contamination in RNA preparations was tested by including RNA samples that had not been reverse transcribed. Amplifications were carried out in an Eppendorf Master Cycler (Dr. Vaudaux AG, Schonenbuch, Switzerland) under the following conditions: denaturation for 3 minutes at 94°C followed by cycles of 30 seconds denaturation at 94°C, 30 seconds annealing at the primer-specific temperature, and 45 seconds elongation at 72°C. Amplifications of IL-1Rrp2 (primer pair A) and IL-1F8 were performed with 45 cycles, whereas amplification of β-actin was performed with 25 cycles. PCR products were visualized on 2% agarose gels containing ethidium bromide. All PCR products were cloned into the pCRII-TOPO^® ^vector (Life Technologies) and their identity was checked by sequencing.

### Quantitative real-time polymerase chain reaction analysis

Expression of 28S ribosomal RNA, human IL-1Rrp2 (primer pair B), IL-1F8 and IL-1β mRNAs was determined by quantitative real-time PCR on reverse-transcribed samples using a light cycler (Roche Diagnostics, Rotkreuz, Swizerland) with the DNA Master SYBR green I or Fast Start DNA Master SYBR green I kits as appropriate. Template cDNAs (2 μl) were amplified in a typical 10 μl PCR reaction containing 0.25 μmol/l of the respective primers (Table 1). The absence of DNA contamination in RNA preparations was tested by including RNA samples that had not been reverse transcribed. Primer sequences and conditions for each PCR reaction are detailed in Table 1. Expression of IL-1Rrp2, IL-1F8, or IL-1β mRNA was corrected for 28S ribosomal RNA levels.

### Preparation of human IL-1F8 and IL-1F5 recombinant proteins

Recombinant human IL-1F8 and IL-1F5 were produced in *Escherichia coli*, as previously reported [3]. To remove endotoxin contamination, protein samples were treated with polymyxin B-agarose beads (Sigma, Buchs, Switzerland). Moreover, in order to check that the effects of IL-1F8 were due to the protein itself and not to endotoxin contamination, in some experiments IL-1F8 was heat-inactivated at 95°C for 5 minutes before use. Commercial human recombinant IL-1F8 (R&D Systems) was used for comparison in some experiments and similar data were obtained with our recombinant protein and with commercial IL-1F8.

### Determination of IL-6, IL-8 and nitric oxide levels

For determination of IL-6, IL-8 and nitric oxide production, hSFs and primary hACs were plated in 96-well plates at a density of 40,000 cells per well. Cells were treated for 48 hours with the indicated concentrations of IL-1β, IL-1F5 and/or IL-1F8. In some experiments cells were preincubated for 1 hour with anti-IL-1Rrp2 antibodies (10 μg/ml) before stimulation with IL-1F8 or IL-1β. Levels of IL-6 and IL-8 in cell supernatants, as well as IL-6 levels in human serum, were assessed using enzyme-linked immunosorbent assay (ELISA) kits from R&D Systems. Production of nitric oxide was assessed, as previously described [24], by the Griess reaction using a NaNO_2 _standard.

### Human and mouse tissue samples

Synovial biopsies from patients with OA or inflammatory arthritides (two patients with RA, one with Lyme disease, one with sacroid arthritis and one with seronegative arthritis) were obtained by knee arthroscopy. All samples were immediately frozen in liquid nitrogen. Samples were obtained after appropriate informed consent, and their use for research was approved by the Ethics Committee of the University Hospital of Geneva.

For induction of CIA, male DBA/1 mice aged between 8 and 10 weeks (Janvier, Le Genest-St-Isle, France) were immunized with 100 μg native bovine collagen type II (Morwell Diagnostics, Zumikon, Switzerland), emulsified in complete Freund's adjuvant containing 5 mg/ml *Mycobacterium tuberculosis *(Difco, Basel, Switzerland), by intradermal injection at the base of tail. On day 21, a booster injection of 100 μg collagen type II in incomplete Freund's adjuvant was given at the base of the tail. From day 15 after the first immunization onward, mice were examined daily for the onset of clinical arthritis. Mice were killed at various time points after disease onset and arthritic knees were removed and immediately frozen in liquid nitrogen. Control knees were obtained from naïve DBA/1 mice and from immunized DBA/1 mice without clinical signs of arthritis. Skin was obtained from phorbol 13-myristate 12-acetate (PMA; Sigma, Buchs, Switzerland) treated and control DBA/1 mice. PMA (1 μg in 200 μl acetone), or acetone (200 μl) for control mice, was applied to the dorsal surfaces of shaved mice. Application of PMA plus acetone or acetone alone was repeated 24 and 48 hours later. Mice were killed 48 hours after the last application and small pieces of skin were immediately frozen in liquid nitrogen. Institutional approval was obtained for all animal experiments.

### Determination of IL-1F8 protein levels by enzyme-linked immunosorbent assay

For determination of IL-1F8 production, hSFs and primary hACs were plated in 96-well plates at a density of 40,000 cells per well. Cells were treated (or not treated) for 48 or 72 hours with 1 ng/ml IL-1β before supernatants were collected. Human and mouse tissue samples were homogenized in ice-cold TNT buffer (50 mmol/l Tris [pH 7.4], 150 mmol/l NaCl, 1 mmol/l PMSF, 0.5% Triton X-100) and the lysates were cleared by centrifugation at 13,000 rpm (4°) for 15 minutes. Protein concentration in the lysates was assessed using the Biorad DC protein assay kit (Bio-Rad Laboratories, Hercules, CA, USA). Human serum and synovial fluid samples were obtained from patients with RA or OA, and control serum was obtained from healthy blood donors. Serum samples from patients with septic shock were kindly provided by Dr Pugin (Department of Intensive Care, University Hospital of Geneva, Geneva, Switzerland). For determination of IL-1F8 levels in culture supernatants, human tissue lysates, human serum or synovial fluids, 96-well plates were coated with a polyclonal anti-human IL-1F8 antibody (R&D Systems), diluted to 1 μg/ml in phosphate-buffered saline (PBS). Wells were then washed with PBS containing 0.05% Tween-20 and blocked with 1% bovine serum albumin (BSA) in PBS. Samples were applied to the wells for two hours at room temperature. After washing, a biotin-conjugated polyclonal anti-human IL-1F8 antibody (R&D Systems) was added at a dilution of 1/1000 in PBS and 1% BSA, and incubated for 2 hours at room temperature. Bound antibody was detected by incubation with streptavidin-horseradish peroxidase (dilution 1/1000 in PBS and 1% BSA) for 20 minutes. Colour was developed using tetramethylbenzidine and H_2_O_2_, the reaction was stopped with H_2_SO_4 _2N, and optical density was assessed at 450 nm. Recombinant human IL-F8 was used for the standard curve. To detect mouse IL-1F8, a similar ELISA was set up using a polyclonal anti-mouse IL-1F8 antibody, a biotin-conjugated polyclonal anti-mouse IL-1F8 antibody and recombinant mouse IL-1F8 (R&D Systems). The detection limit of these assays was 19 pg/ml.

### Statistical analysis

The significance of differences was calculated by analysis of variance or Mann-Whitney test as appropriate. A difference between experimental groups was considered statistically significant when the *P *value was below 0.05.

## Results

As a first approach to investigate expression of new IL-1 family members during arthritis, we examined IL-1F5 to IL-1F10 mRNA expression by RT-PCR in joints of mice with CIA and in synovial biopsies from patients with RA or OA. IL-1F8 was the only new IL-1 family member for which we detected mRNA expression both in human synovial biopsies and in mouse joints. In addition, we also observed IL-1F9 mRNA expression in mouse joints, whereas expression of IL-1F6 and IL-1F7 mRNA was detected in some human synovial samples (data not shown).

**Figure 1:**
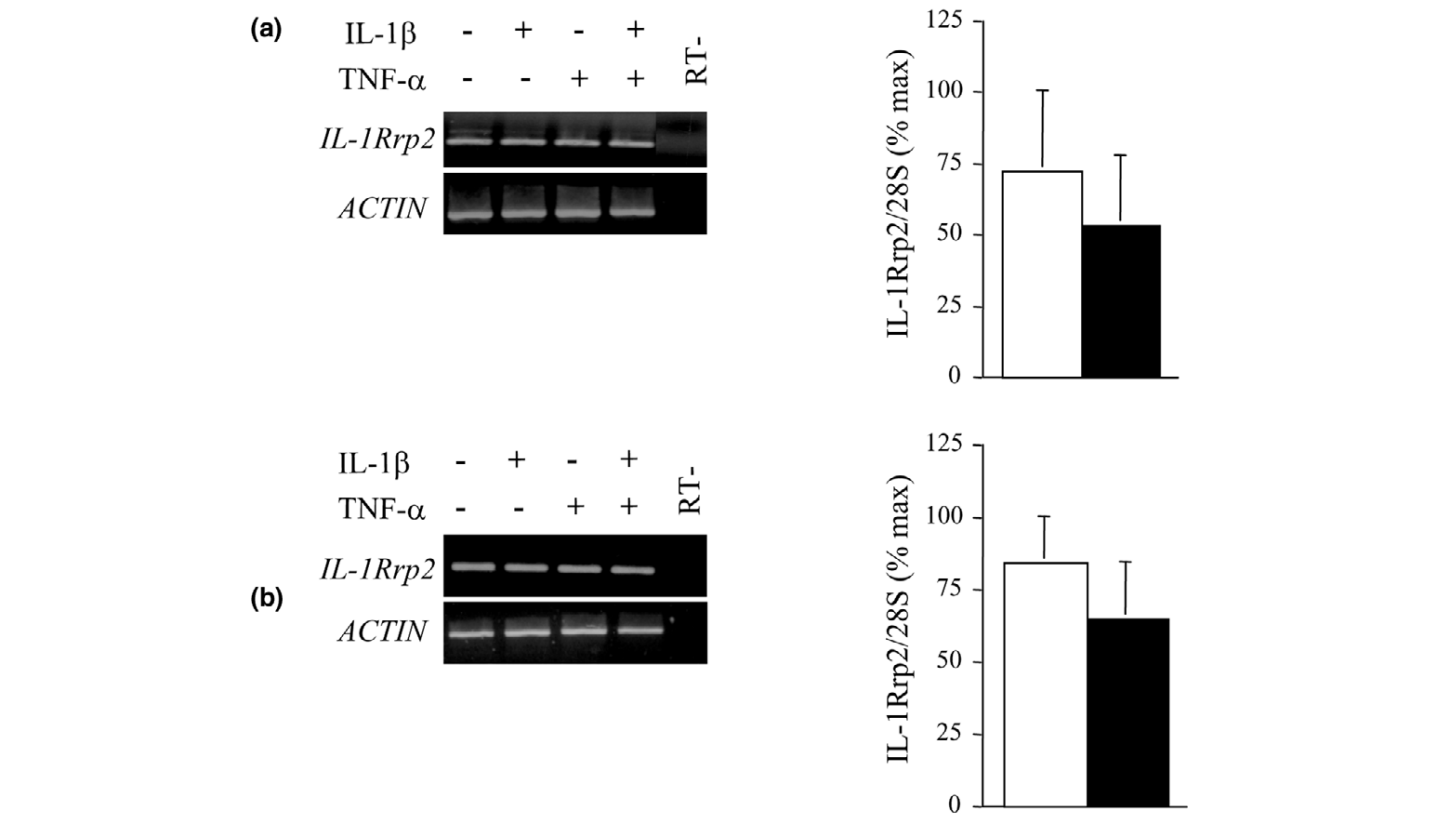
IL-1Rrp2 expression by hSFs and hACs. The left panels show a RT-PCR analyses of IL-1Rrp2 expression by **(a) **hSFs and **(b) **hACs treated or not treated for 8 hours by IL-1β (1 ng/ml) and/or TNF-α (10 ng/ml), as detailed under Materials and method and in Table 1. The images show representative agarose gel electrophoresis of PCR products. The right panels show real-time PCR analysis of IL-1Rrp2 mRNA levels in hSFs and hACs stimulated (black columns) or not stimulated (white columns) for 8 hours with IL-1β (1 ng/ml) and TNF-α (10 ng/ml). The amount of 28S rRNA was monitored as an internal control. The expression of IL-1Rrp2 mRNA was corrected for 28S rRNA levels and the IL-1Rrp2/28S ratios were normalized to the maximal value observed in each experiment, which was set to 100%. The results shown represent the mean ± standard error of data obtained with samples from three (hSFs) or four (hACs) independent cultures. IL, interleukin; IL-1Rrp2, IL-1 receptor related protein 2; hAC, human articular chondrocyte; hSF, human synovial fibroblast; RT-PCR, reverse transcriptase polymerase chain reaction; TNF, tumour necrosis factor.

A recent study [16] reported that IL-1F8 signalling requires the presence of both IL-1Rrp2 and IL-1RAcP. Therefore, we investigated IL-1Rrp2 mRNA expression by hSFs and hACs, because IL-1RAcP expression in these cells has already been reported [25] and is further demonstrated by their well established responsiveness to IL-1β. As shown in Figure 1, both hSFs and hACs expressed basal levels of IL-1Rrp2 mRNA, which were not upregulated by IL-1β and/or TNF-α. In contrast, we did not observe IL-1Rrp2 expression in THP-1 and Jurkat cell lines (data not shown), confirming previous findings [16,25].

**Figure 2:**
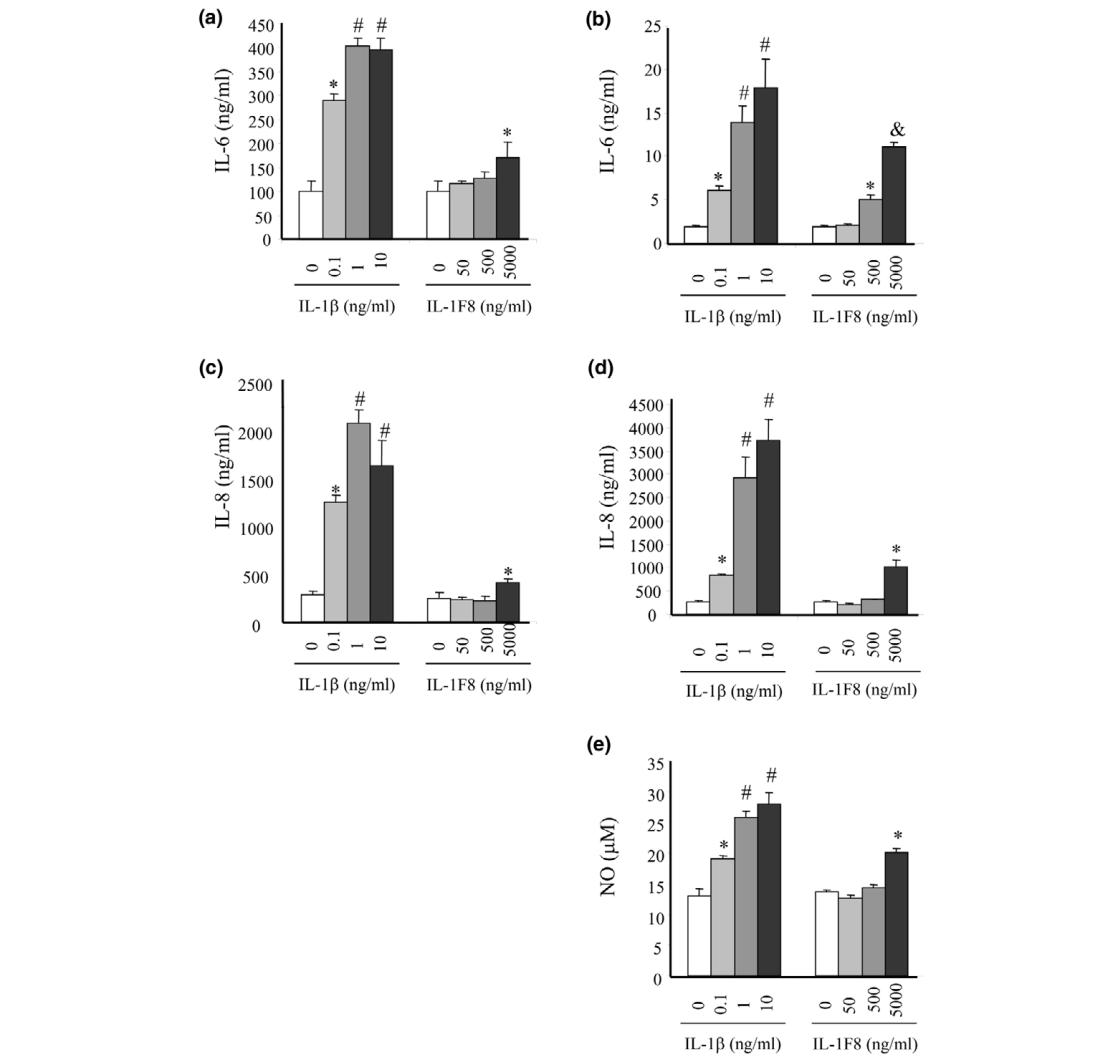
Production of IL-6, IL-8 and nitric oxide by hSFs and hACs: effects of IL-1F8 and IL-1β. Shown is an analysis of the effects of IL-1F8 and IL-1β on production of **(a) **IL-6 and **(c) **IL-8 by hSFs, and of **(b) **IL-6, **(d) **IL-8 and **(e) **nitric oxide by hACs. Cells were treated with the indicated cytokine concentrations for 48 hours, as detailed under Materials and method. *P < 0.05 versus control; ^#^*P *< 0.05 versus 0.1 ng/ml IL-1β; ^&^*P *< 0.05 versus 500 ng/ml IL-1F8, determined using analysis of variance. IL, interleukin; hAC, human articular chondrocyte; hSF, human synovial fibroblast.

**Figure 3:**
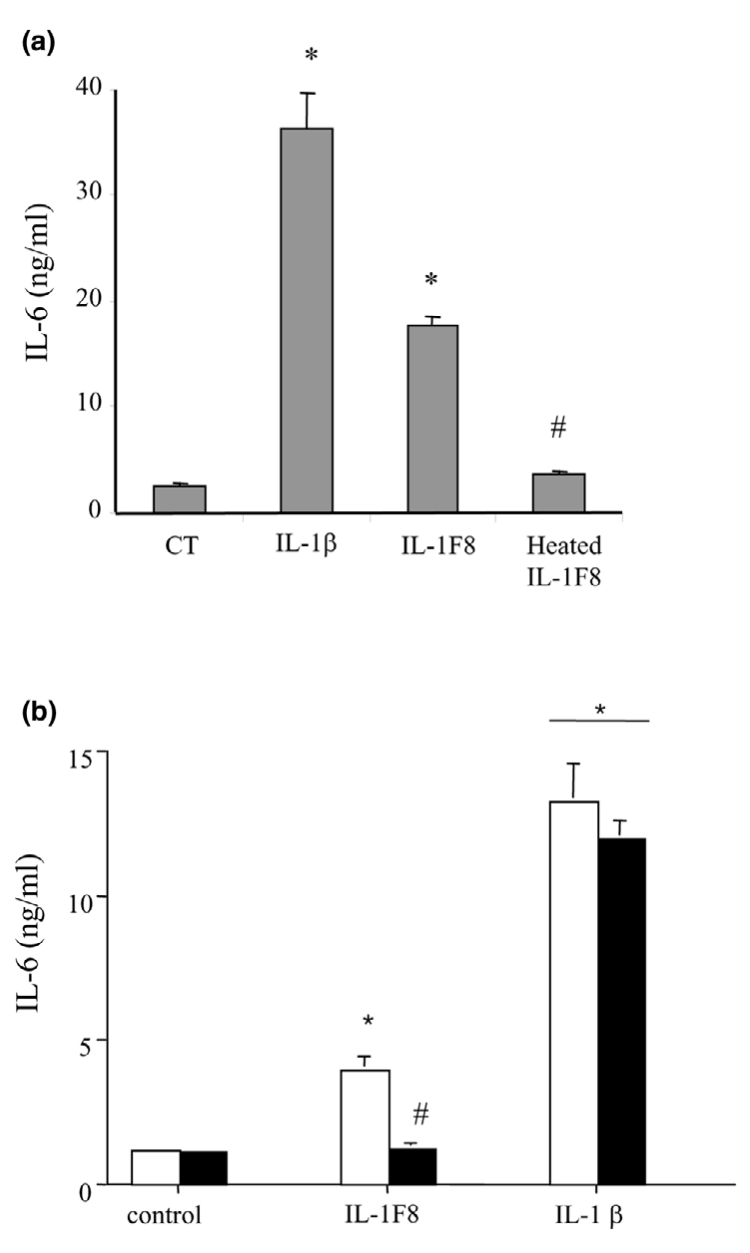
Production of IL-6 by hACs: effects of IL-1β, IL-1F8, heat-inactivated IL-1F8 and anti-IL-1Rrp2 antibodies. **(a) **Analysis of the effects of IL-1β (1 ng/ml), IL-1F8 and heat-inactivated IL-1F8 (5 mg/ml) on IL-6 production by hACs after 48 hours treatment, as detailed under Materials and method. **(b) **Analysis of the effects of anti-IL-1Rrp2 antibodies on IL-6 production by hACs. Cells were stimulated or not (control) for 48 hours with IL-1F8 (5 mg/ml) or IL-1b (1 ng/ml), as indicated, in the presence (black columns) or absence (white columns) of blocking anti-IL-1Rrp2 antibodies (10 mg/ml). *P *< 0.05 versus control; ^#^*P *< 0.05 versus 5 μg/ml IL-1F8, as determined by analysis of variance. hAC, human articular chondrocyte; IL, interleukin; IL-1Rrp2, IL-1 receptor related protein 2.

Because hSFs and hACs express IL-1Rrp2 mRNA, we hypothesized that these cells should be able to respond to IL-1F8 without need for receptor over-expression. As indicated by Figure 2, IL-1F8 stimulated both IL-6 and IL-8 production in hSFs and hACs. The response was stronger in hACs, with a significant increase in IL-6 production with 500 ng/ml of IL-1F8. In addition, IL-1F8 also stimulated nitric oxide production by hACs (Figure 2e), suggesting that its effects might be similar to those exhibited by IL-1β. There was no synergy between IL-1β and IL-1F8 for the stimulation of IL-6 production by hACs, and the effect of 5 μg/ml IL-1F8 was additive with that of low doses of IL-1β (1–10 pg/ml; data not shown). Furthermore, the effects of IL-1F8 were indeed due to the protein itself and not to endotoxin contamination because heat-inactivated IL-1F8 failed to stimulate IL-6 production in hACs (Figure 3a). The effects of IL-1F8 were mediated by IL-1Rrp2 and could be completely blocked in presence of a polyclonal anti-IL-1Rrp2 antibody (Figure 3b). Finally, the reported correlation between IL-1F8 responsiveness and IL-1Rrp2 expression [16] was supported by our observation that C28/I2 and SW1353 'chondrocyte-like' cell lines and human dermal fibroblasts in which levels of IL-1Rrp2 mRNA were very low or absent did not produce IL-6 in response to 5 μg/ml IL-1F8. In contrast, incubation of these cells with 1 ng/ml of IL-1β stimulated IL-6 production (data not shown).

Debets and coworkers [5] reported that IL-1F5 could antagonize the effects of IL-1F9 when it was added at equimolar concentrations; we therefore tested the ability of recombinant human IL-1F5 concentrations from 50 ng/ml to 5 μg/ml to inhibit the effects of 5 μg/ml IL-1F8 on IL-6 production in hACs. In these conditions, antagonism by IL-1F5 of the effects of IL-1F8 was incomplete and not reproducible (data not shown).

**Figure 4:**
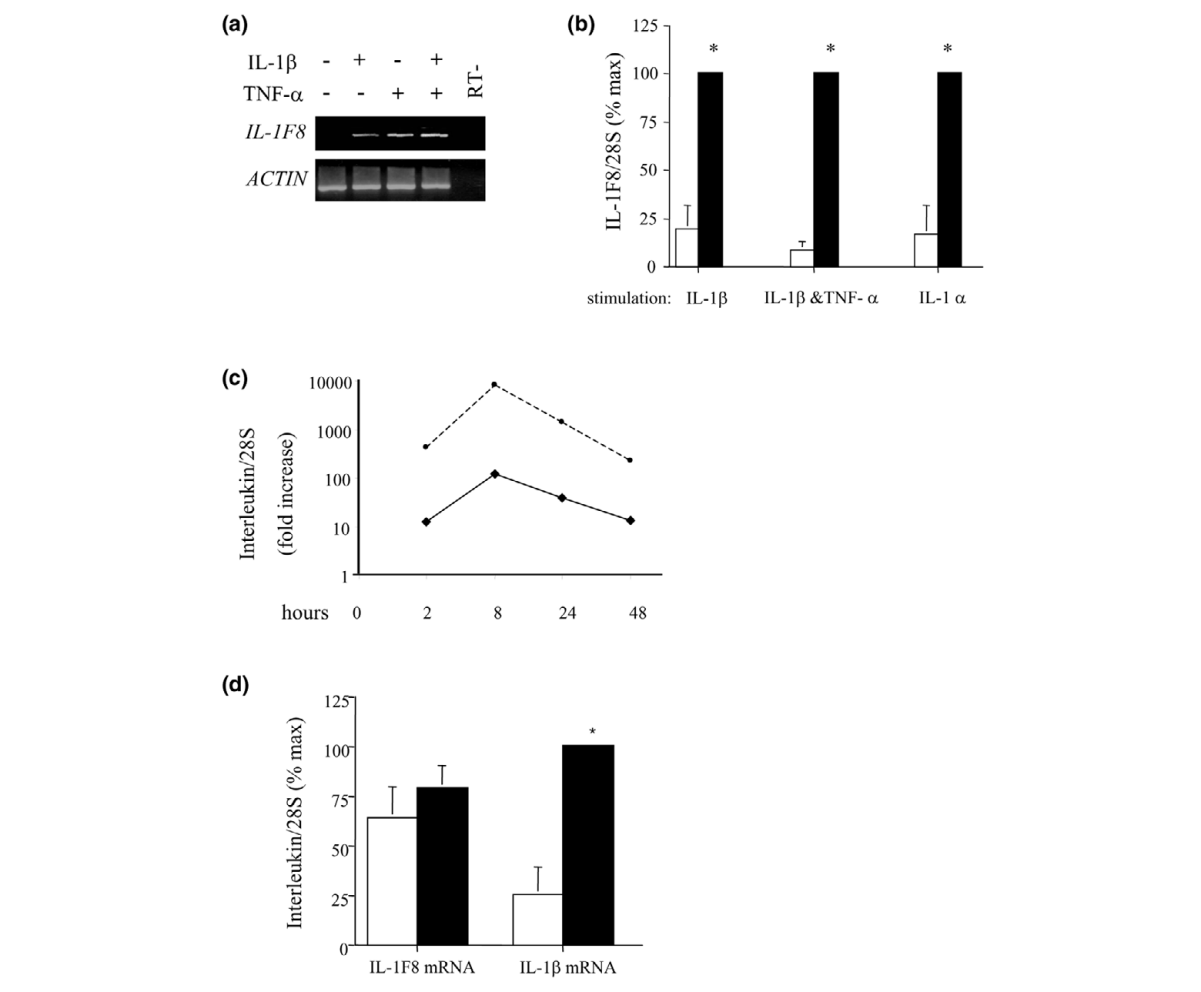
Kinetics of IL-1β and IL-1F8 mRNA production by HSFs and hCAs in response to IL-1 and/or TNF-α. **(a) **Analysis of IL-1F8 mRNA levels in hSFs treated or not treated for 8 hours with IL-1β (1 ng/ml) and/or TNF-α (10 ng/ml), as detailed under Materials and method. A representative agarose gel electrophoresis of PCR products is shown. **(b) **Real-time PCR analysis of IL-1F8 mRNA levels in hSFs stimulated (black columns) or not stimulated (white columns) for 8 hours with IL-1β (1 ng/ml) alone, IL-1β (1 ng/ml) plus TNF-α (10 ng/ml), or IL-1α (1 ng/ml) alone, as indicated. The amount of 28S rRNA was monitored as an internal control. The expression of IL-1F8 mRNA was corrected for 28S rRNA levels and the IL-1F8/28S ratios were normalized to the maximal value observed in each experiment, which was set to 100%. The results shown represent the mean ± standard error of data obtained with samples from three independent cultures. *P < 0.05 versus, as determined by analysis of variance. **(c) **Fold increase (after correction for 28S RNA levels) in IL-1β (dashed line) and IL-1F8 (solid line) mRNA levels after treatment of hSFs with 1 ng/ml IL-1β for the indicated times, as revealed by real-time PCR analysis. Basal IL-1F8/28S and IL-1β/28S levels were respectively 6.4 and 20 (arbitrary units). **(d) **Real-time PCR analysis of IL-1F8 and IL-1β mRNA levels in hACs stimulated (black columns) or not stimulated (white columns) with IL-1β (1 ng/ml) and TNF-α (10 ng/ml) for 8 hours. The amount of 28S rRNA was monitored as an internal control. The expression of IL-1F8 and IL-1β mRNA was corrected for 28S rRNA levels and the IL/28S ratios were normalized to the maximal value observed in each experiment, which was set to 100%. The results shown represent the mean ± standard error of data obtained with samples from six independent cultures. *P < 0.05 versus, as determined by analysis of variance. hAC, human articular chondrocyte; hSF, human synovial fibroblast; IL, interleukin; RT-PCR, reverse transcriptase polymerase chain reaction; TNF, tumour necrosis factor.

We then screened various cell types present in the inflamed joint for endogenous IL-1F8 mRNA expression *in vitro*. By RT-PCR, IL-1F8 expression was observed in hSFs when they were treated for eight hours with IL-1β, TNF-α, or both (Figure 4a). By real-time PCR, we confirmed increased IL-1F8 mRNA expression after 8 hours stimulation of hSFs with IL-1β alone, IL-1β plus TNF-α, or IL-1α alone (Figure 4b). The increase in IL-1F8 mRNA levels was strongest with stimulation by 1 ng/ml IL-1β, as compared with 0.1 and 10 ng/ml (data not shown). The steady state levels of IL-1F8 mRNA expression peaked at 8 hours (Figure 4c) in response to 1 ng/ml IL-1β, which is similar to the time course of induction of endogenous IL-1β mRNA by IL-1β in hSFs. In hACs expression of IL-1F8 mRNA was constitutive and IL-1F8 levels were not affected by stimulation of cells with IL-1β and TNF-α for 8 hours, whereas this treatment consistently induced IL-1β gene expression (Figure 4d). We also observed that the THP-1 monocyte cell line and Jurkat T-cell line expressed basal levels of IL-1F8 mRNA, but real-time PCR experiments failed to detect IL-1F8 mRNA upregulation in response to various stimuli, including IL-1β, TNF-α, IL-4 and PMA (data not shown).

**Figure 5:**
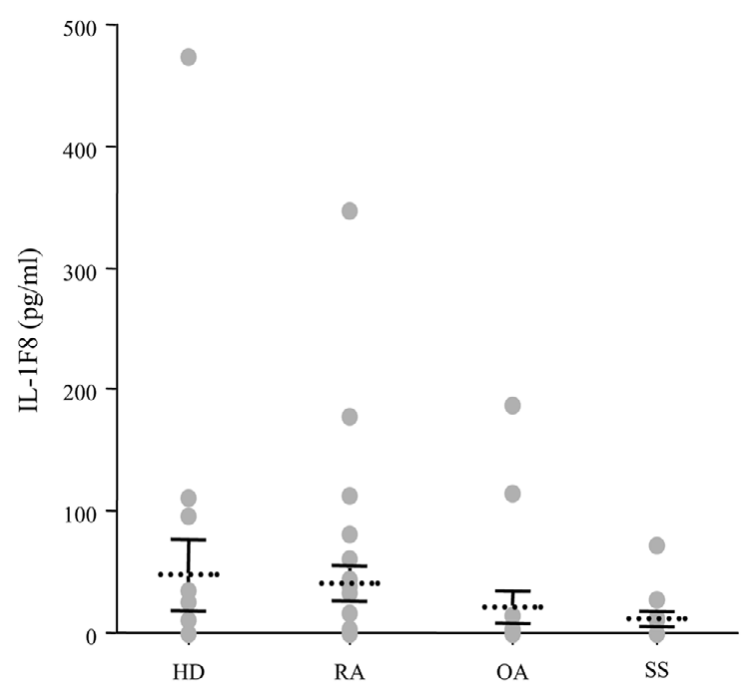
IL-1F8 protein levels in control individuals, patients with RA, OA and septic shock. Shown are serum IL-1F8 protein levels in healthy donors (*n *= 16), patients with RA (*n *= 28) or OA (*n *= 16) patients, and patients with septic shock (*n *= 12), as determined by ELISA. Individual values (grey dots) and mean (stippled lines) ± standard error (black lines) are shown. Differences between the groups were not significant. ELISA, enzyme-linked immunosorbent assay; IL, interleukin; OA, osteoarthritis; RA, rheumatoid arthritis.

Next, we assessed IL-1F8 protein levels by ELISA in culture supernatants of hSFs and hACs stimulated (or not stimulated) with IL-1β for 48 or 72 hours. IL-1F8 protein levels were below the limit of detection of the ELISA (19 pg/ml) in all samples. We also measured IL-1F8 and IL-1β protein expression in synovial biopsies of patients with various inflammatory arthritides or OA (Table 2). As expected, IL-1β levels were elevated in inflammatory arthritis synovial biopsies. On the contrary, IL-1F8 protein levels were not increased in inflamed joint tissue. IL-1F8 levels measured in synovial fluids were consistently similar to or lower than those in matched serum samples obtained from OA (*n *= 4) and RA (*n *= 4) patients, suggesting that the joint itself is not a major source of IL-1F8 (data not shown). We thus analyzed IL-1F8 levels in the serum of 28 RA and 16 OA patients, 16 healthy controls, as well as 12 patients with septic shock. Serum levels of IL-1F8 were not different between the groups, although they tended to be higher in healthy donors and in RA patients than in patients with OA and septic shock (Figure 5). In healthy donors, RA and OA patients, IL-1F8 levels did not correlate with serum IL-6 levels, which were used as a marker of inflammation (data not shown). Interestingly, we nevertheless observed high IL-1F8 levels (>50 pg/ml) in three out of 16 healthy donors, six out of 28 RA patients, two out of 16 OA patients, and one out of 12 septic patients (Figure 5).

We also examined serum IL-1F8 levels in RA patients (*n *= 9) before and after anti-TNF treatment. Serum IL-1F8 levels remained unchanged in RA patients after 8–36 weeks of anti-TNF treatment, independently of the amelioration of clinical symptoms (data not shown). Using a similar ELISA for mouse IL-1F8, we measured IL-1F8 and IL-1β protein expression in knees of mice with or without CIA (Table 2). Again, although IL-1β levels were elevated in mouse joints during CIA, IL-1F8 levels were not. Serum IL-1F8 levels were below the limit of detection of the ELISA in the serum of mice with CIA between 1 and 23 days after the onset of arthritis, as well as in control naïve mice and in type II collagen-immunized mice exhibiting no clinical signs of arthritis. Finally, in contrast to our observation in joints, we detected very high IL-1F8 protein levels in mouse skin, which were further increased during PMA-induced skin inflammation (Table 2). IL-1β levels increased in parallel, although the amounts of protein detected in the skin were much lower for IL-1β than for IL-1F8.

## Discussion

There is currently a huge body of evidence indicating that IL-1α, IL-1β, IL-1Ra and IL-18 are involved at some level in the pathophysiology of RA (for review see [26,27]). We thus hypothesized that some of the six new members of the IL-1 family might also play a role during RA. Therefore, we sought to investigate the effects and the expression of new IL-1 family members in joint cells.

Investigation of the *in vitro *effects of recombinant human IL-1F8 revealed a direct correlation between IL-1Rrp2 expression and IL-1F8 responsiveness. Our results further support findings indicating that IL-1Rrp2 is required for IL-1F8 signalling [5,16]. Both hSFs and hACs produced inflammatory mediators in response to IL-1F8, and stimulation of IL-6 and IL-8 production was somewhat stronger in hACs than in hSFs. To our knowledge, the present study is the first to report responsiveness of nontransfected, primary cells to one of the recently discovered IL-1 family members. In contrast, Wang and coworkers [28] recently failed to detect an effect of recombinant IL-1F8 on mixed glial cell cultures, which might be related to low levels of IL-1Rrp2 expression. Indeed, the correlation between IL-1F8 responsiveness and IL-1Rrp2 expression is supported by our observation that various cell lines in which levels of IL-1Rrp2 mRNA were low did not produce IL-6 in response to 5 μg/ml IL-1F8. In addition, our results indicate that amounts of recombinant IL-1F8 required to stimulate hSFs and hACs are higher than those of IL-1β, which is in agreement with recent work reported by Towne and coworkers [16]. Those authors reported significant stimulatory effects at similar IL-1F8 concentrations as in the present study (500–5,000 ng/ml).

The need for high concentrations of recombinant IL-1F8 is not understood, and thus far no biological effect of any of the new IL-1 family members has been reported at below about 10^-7 ^mol/l, as compared with about 10^-11 ^mol/l for IL-1β and about 10^-9 ^mol/l for IL-18. Interestingly, we recently observed that transfection of IL-1Rrp2 expressing C20A4 chondrocytic cells with an expression vector for human IL-1F8, which led to the production of moderate quantities of IL-1F8 (50–200 pg/ml in culture supernatants after 48 hours), efficiently induced IL-6 secretion in these cells, as compared with empty vector transfected control cells (GP, FM and CG; unpublished observations). These observations suggest that endogenously expressed IL-1F8 is active at much lower doses than recombinant IL-1F8, although the reason for this discrepancy is still unknown and is currently under investigation. One hypothesis is that post-translational modifications of the IL-1F8 protein might be important for its biological activity and might be lacking in recombinant IL-1F8 produced in *E. coli*. Levels of IL-1F8 detected for RA patients ranged up to 347 pg/ml in serum and up to 176 pg/ml in synovial fluid, and according to our observations in C20A4 cells such concentrations of endogenously produced IL-1F8 might be sufficient to trigger biological effects in joint cells.

There is some controversy concerning the putative antagonist effects of IL-1F5 [5,16]. Debets and coworkers [5] have shown that IL-1F5 inhibits IL-1F9 induced nuclear factor-κB activation in Jurkat T cells overexpressing IL-1Rrp2 [5], but Towne and coworkers [16] did not observe consistent inhibitory effects of IL-1F5 on IL-1F6-, IL-1F8-, or IL-1F9-induced activation of nuclear factor-κB in the same cells. Although in some experiments we observed antagonistic effects of IL-1F5 on the inflammatory action of IL-1F8 on hACs and hSFs, this antagonism was inconsistent and incomplete. We currently have no explanation for these nonreproducible effects. The use of primary cultures may account for such findings in our study but not in that of Towne and coworkers [16], who used cell lines. It is also possible that recombinant IL-1F5 lacks conformational stability or post-translational modification, and that this may alter its activity. The possible role played by IL-1F5 therefore remains unknown.

Investigation by RT-PCR of their expression in joints of mice with CIA and in synovial tissue from patients with RA revealed that, among the newly cloned IL-1 family members, only IL-1F8 was expressed in both mouse and human joints. Quantitative PCR experiments demonstrated a significant upregulation of IL-1F8 mRNA levels in cultured hSFs in response to IL-1β and/or TNF-α. In contrast, IL-1F8 mRNA expression was constitutive in hACs and was not affected by inflammatory stimuli. Similarly, although monocyte and T-lymphocyte cell lines express IL-1F8 mRNA to some extent, IL-1F8 levels were not increased in response to a panel of stimuli. It has been reported that T cells, either stimulated with anti-CD3 and/or anti-CD28 or left unstimulated, do not express IL-1F8 mRNA, whereas lipopolysaccharide-treated monocytes do [10]. Despite IL-1F8 mRNA expression, IL-1F8 protein expression was below the limit of detection of our assay in hSF and hAC culture supernatants. In human OA and normal mouse joint tissue, IL-1F8 protein expression levels were similar to those of IL-1β. However, although IL-1β protein levels were increased in inflamed joints, IL-1F8 levels were not. Interestingly, a very different situation applied to mouse skin samples, in which IL-1F8 levels were very high and further increased with inflammation. Furthermore, IL-1F8 levels in synovial fluids were similar to or lower than those measured in matched serum samples, suggesting that the joint itself is not a major source of IL-1F8. Indeed, in the case of IL-6, for instance, which is produced in the joint, synovial fluid concentrations are 100-fold to 1000-fold higher than those measured in serum [29]. Serum levels of IL-1F8 did not differ between healthy donors, and patients with RA, OA and septic shock, and did not correlate with inflammatory status. Interestingly, however, we observed high IL-1F8 levels in several serum samples in all of these groups. The cause of such high serum IL-F8 levels and the source of circulating IL-1F8 are as yet unknown.

## Conclusion

IL-1F8 exerts proinflammatory effects in primary human joint cells. However, although IL-1F8 mRNA is expressed in hSF and hAC, joint cells are not a major source of IL-1F8 protein. Joint and serum IL-1F8 protein levels did not correlate with inflammation, but IL-1F8 was elevated in some human serum samples tested, including several samples from RA patients. It remains to be determined whether, in some cases, circulating IL-1F8 can contribute to joint inflammation in RA.

## Abbreviations

BSA = bovine serum albumin; CIA = collagen-induced arthritis; ELISA = enzyme-linked immunosorbent assay; FCS = foetal calf serum; hAC = human articular chondrocyte; hSF = human synovial fibroblast; IL = interleukin; IL-1Ra = IL-1 receptor antagonist; IL-1RacP = IL-1 receptor accessory protein; IL-1Rrp2 = IL-1 receptor related protein 2; OA = osteoarthritis; PBS = phosphate-buffered saline; PMA = phorbol 13-myristate 12-acetate; RA = rheumatoid arthritis; RT-PCR = reverse transcriptase polymerase chain reaction; TNF = tumour necrosis factor.

## Competing interests

The authors declare that they have no competing interests.

## Authors' contributions

DM, GP, FM and DT-A performed the experiments concerning the *in vitro *effects of IL-1F8, as well as the mRNA and protein expression studies. SB, TD and MN collected and provided human tissue, synovial fluid and serum samples. JLB and MJHN produced the recombinant IL-1F proteins. DM, GP, SB, PAG, MJHN and CG participated in the design of the study, data analysis, and drafting and reviewing of the manuscript. All authors read and approved the final manuscript.

## Figures and Tables

**Table 1:** Summary of primers used

cDNA	Forward and reverse primers	Ta (°C)	Product (bp)	GenBank
IL-1Rrp2 (A)	F: 5'-AGCAAAATCCCAGTGTCCAAA-3'	60	291	[AF284434]
	R: 5'-ACCCAAAACACAACTCTTCGG-3'			
IL-1Rrp2 (B)	F: 5'-AGCAAAATCCCAGTGTCCAAA-3'	60	147	[AF284434]
	R: 5'-GGTTTACATGTATTCTATGACAG-3'			
IL-1F8	F: 5'-ACCAAGGAGAGAGGCATAACTAAT-3'	60	147	[NM173178]
	R: 5'-AGTGAACTCAGTCGCATAATGATC-3'			
IL-1β	F: 5'-GCTGAGGAAGATGCTGGTTC-3'	57	146	[NM000756]
	R: 5'-GTGATCGTACAGGTGCATCG-3'			
β-actin	F: 5'-CCAAGGCCAACCGCGAGAAGATGAC-3'	55	579	[M10277]
	R: 5'-AGGGTACATGGTGGTGCCGCCAGAC-3'			
28S	F: 5'-TTGAAAATCCGCGGGAGA-3'	54	100	[U13369]
	R: 5'-ACATTGTTCCAACATGCCAG-3'			

Shown are the primer sequences, annealing temperatures (Ta), lengths of the corresponding PCR products, and GenBank accession numbers of the DNA sequences. F, forward; IL, interleukin; IL-1Rrp2, IL-1 receptor related protein 2; R, reverse; PCR, polymerase chain reaction.

**Table 2:** IL-1F8 protein levels in mouse and human joint samples and in mouse skin

Samples	Patients/animals	IL-1F8 (pg/mg protein)	IL-1β (pg/mg protein)
Human synovium	OA (*n *= 4)	3.7 ± 1.4	3.6 ± 0.3
	Inflammatory arthritis (*n *= 5)	3.9 ± 1.0	11.6 ± 5.7^a^
Mouse joint	Naïve (*n *= 1)	2.8	2.3
	Nonarthritic (*n *= 1)	2.7	4.5
	CIA early^b ^(*n *= 3)	2.2 ± 0.3	16.6 ± 1.2^c^
	CIA late^b ^(*n *= 3)	4.5 ± 1.4	8.8 ± 0.8
Mouse skin	Control (*n *= 3)	761.7 ± 313.4	16 ± 3
	PMA (*n *= 3)	14569.1 ± 3632.5^d^	28 ± 3^d^

IL-1F8 protein levels were determined using enzyme-linked immunosorbent assay. ^a^*P *< 0.05 versus osteoparthritis (OA), as assessed using the Mann-Whitney test. ^b^Collagen-induced arthritis (CIA) early: days 1–7 after the onset of arthritis; CIA late: days 8–21 after onset of arthritis. ^c^*P *< 0.05 versus late CIA, as assessed using analysis of variance. ^d^*P *< 0.05 versus control, as assessed using analysis of variance.
